# Effects of Probiotic Supplementation on TGF-β1, TGF-β2, and IgA Levels in the Milk of Japanese Women: An Open-Label Pilot Study

**DOI:** 10.3389/fnut.2019.00128

**Published:** 2019-09-03

**Authors:** Tomoki Takahashi, Hirofumi Fukudome, Hiroshi M. Ueno, Shiomi Watanabe-Matsuhashi, Taku Nakano, Toshiya Kobayashi, Kayoko Ishimaru, Atsuhito Nakao

**Affiliations:** ^1^Research and Development Department, Bean Stalk Snow Co., Ltd., Saitama, Japan; ^2^Department of Immunology, Faculty of Medicine, University of Yamanashi, Yamanashi, Japan

**Keywords:** TGF-β, IgA, cytokine, probiotics, human milk

## Abstract

**Background:** Dietary probiotics supplementation in lactating mothers may help prevent allergic disease in infants. However, owing to a lack of consistency in nutritional and safety outcomes associated with probiotics, this topic remains controversial.

**Methods:** In this open-label pilot trial conducted between April 2013 and December 2013, we evaluated the safety of probiotic supplementation with 5 × 10^9^ CFU of Lactobacillus casei LC5, 5 × 10^9^ CFU of Bifidobacterium longum BG7, and 2 × 10^8^ CFU of Bacillus coagulans SANK70258 in lactating women who exhibited allergies for 2 months (1–3 months postpartum); we also evaluated the effects of probiotic supplementation on transforming growth factor-β (TGF-β) and immunoglobulin A (IgA) levels in human milk. Participants self-selected to join the probiotic (*n* = 41; age [median (interquartile range [IQR]), y] 33 [27–39], body mass index [BMI] [median (IQR), kg/m^2^] 21.8 [19.5–22.8]) or no supplementation control group (*n* = 19; age [median (IQR), y] 33 [23–43], BMI [median (IQR), kg/m^2^) 19.6 [18.4–22.1]). Probiotics (three tablets) received were taken as daily supplements. Milk samples were collected at 1, 2, and 3 months postpartum, and TGF-β1, TGF-β2, and IgA levels were measured.

**Results:** No adverse effects were observed in the probiotic group, according to the self-recorded diary during the study period. Milk IgA decreased with increasing postpartum months in both groups. In contrast, TGF- β1 and β2 were not affected by lactation periods, and showed different patterns over time between the two groups. TGF-β1, TGF-β1, and IgA levels were significantly correlated at baseline (respectively *p* < 0.05). However, the correlation between TGF-β1 and IgA became non-significant by the end of the intervention (*p* = 0.063).

**Conclusion:** Altogether, probiotic supplementation was tolerated with respect to no dropout and 91.5% adherence. Although probiotic supplementation might affect human milk TGF-β levels, a positive effect of probiotic supplementation was not entirely supported. Future placebo-controlled studies are needed to further support the efficacy and safety of probiotic supplementation.

**Clinical Trial Registration:** www.umin.ac.jp/ctr/, identifier: UMIN000036059.

## Introduction

Allergic diseases are a common public health problem and economically impact the healthcare systems of many countries. Atopic dermatitis is a type of eczema and the most prevalent allergic disorder of the skin, accounting for more than 20% of allergies in children; these include diseases such as food allergies, bronchial asthma, and allergic rhinitis (1, 2). The mean global prevalence of allergic diseases in children has increased by 0.5% annually over the past decades (3). Accordingly, the dietary management of allergies in infants and children may be a promising option for the prevention of these diseases (4).

Probiotic supplementation has been proposed as an intervention for the reduction of eczema risk in infants (5). Maternal supplementation with probiotics, either in pregnancy or during lactation, affects fetal immune parameters and immune markers in breast milk, including transforming growth factor-β (TGF-β) and immunoglobulin A (IgA) (6–8). TGF-β is a key immunoregulatory cytokine with four isoforms (TGF-β1, TGF-β2, TGF-β3, and TGF-β4) in mammals. The TGF-β1 isoform serves as an immunoregulatory cytokine involved in the mucosal immune system; TGF-β2 is more abundant and similarly has immunoregulatory roles but is less studied in breast milk compared to TGF-β1 (9). TGF-β3 and TGF-β4 were respectively identified as effectors of mammary gland involution during the initial phases and as endometrial bleeding associated factors; however, their immunological functions have not been clarified (10, 11). TGF-β also promotes IgA production as well as oral tolerance toward cow's milk. Secretory IgA is a major immunoglobulin found in epithelial tissues that serves as a primary defense agent against pathogenic microorganisms and enteric toxins in the gastrointestinal and respiratory tracts. Although no consistent association has been reported between total and food-specific IgA levels in breast milk and the progress of allergic disease in older children, lower levels of these IgAs are present in the colostrum and breast milk of mothers of offspring who eventually manifest cow's milk allergy (12).

Importantly, the effects of probiotics seem to be strain-specific and dose-dependent, and the quality control process can influence the associated clinical outcomes and result in pathogen contamination (13). A previous report showed that a mixture of different probiotic genera, *Bifidobacterium* and *Lactobacillus*, might be more effective in preventing allergic disease of infants than a single genus; in this report, the subjects were supplemented with ≤10^9^ CFU in many trials (14). On the other hand, an ideal effective combination for the effect of probiotic species and strains has not been clarified. *L. casei* is a Gram-positive bacterium that is naturally present in the human and animal gastrointestinal and mouth organs, and the LC5 strain was isolated from cheese [(15), personal communication]. *B. longum* is a Gram-positive bacterium that inhabits the human gastrointestinal tract, and the BG7 strain was isolated from infant feces (16). *B. coagulans* is a Gram-positive, spore-forming bacterium that is found abundantly, and the SANK70258 strain was isolated from green malt (17). These strains have long histories of consumption, and they are commonly consumed and available for dietary probiotics. Thus, a mixture of different probiotic strains can affect the immune components in human milk. Here, we developed a combination of three commercially available probiotic strains—*Lactobacillus casei* LC5, *Bifidobacterium longum* BG7, and *Bacillus coagulans* SANK70258—and conducted an open-label pilot study to examine the safety and longitudinal effects of the mixed probiotic strains on TGF-β and IgA levels in the breast milk of Japanese women. Moreover, from the viewpoint of industrial application, consumer acceptance, and adherence of such supplement should be also considered. Therefore, the present study also investigated the preference and adherence of this probiotic supplementation in lactating mothers with a history of allergic disease.

In a prospective birth cohort of lactating mothers with allergies, mothers were voluntarily allocated to probiotic supplementation or no supplementation control groups for a 2-month period. We then collected breast milk samples during the intervention period and measured the levels of TGF-β and IgA.

## Materials and Methods

### Participants

The present study was conducted as an open trial to evaluate the effects of probiotic supplementation on TGF-β1, TGF-β2, and IgA in human breast milk. We recruited 63 lactating women and informed them of the content and intention of the study. In this open-label pilot trial, conducted between April 2013 and December 2013, 60 healthy lactating women from the Tokyo metropolitan area volunteered to participate through Gonohashi Obstetrics and Gynecology Hospital (Tokyo, Japan). The inclusion criteria were: (i) lactating, (ii) 30–59 days postpartum, and (iii) a history of allergy at recruitment. The maternal allergy history was determined based on a reported clinical history of atopic eczema, asthma, or allergic rhinitis. The exclusion criteria were: (i) clinical evidence of chronic illness or gastrointestinal disorder, (ii) routine use of any other dietary supplements containing probiotics and fiber, and (iii) smoking at recruitment. Consumption of fermented foods, such as dairy foods, fermented soybeans including miso and natto, and fermented vegetables including leafy vegetables and kimuchi, were not included in the exclusion criteria.

### Probiotic Supplementation

After they consented to participate, women selected to join either the probiotic group or the no supplementation control group. Three women dropped out of the study due to the lack of informed consent, leaving 60 women enrolled in either the probiotic (*n* = 41) or control (*n* = 19) groups. The probiotic group received three probiotics—*Lactobacillus casei* LC5 (CJ Japan Co., Ltd, Tokyo, Japan), *Bifidobacterium longum* BG7 (CJ Japan Co., Ltd, Tokyo, Japan), and *Bacillus coagulans* SANK70258 (Mitsubishi-kagaku Foods Co., Ltd, Tokyo, Japan)—for a period of about 2 months from 1 to 3 months postpartum. The probiotic group received daily supplements of 5 × 10^9^ CFU of *L. casei* LC3, 5 × 10^9^ CFU of *B. longum* BG7, and 2 × 10^8^ CFU of *B. coagulans* SANK70258 in three tablets. The probiotic tablets were prepared by Sunsho Pharmaceutical Co., Ltd. (Fuji, Japan) in 90 tablets (~22.5 g) packs. The composition of the test tablets is shown in Table 1. Tablet ingredients included maltitol, LC5 culture (including corn starch, sodium chloride, and soy protein), BG7 culture (including corn starch, sodium chloride, and soy protein), SANK70258 culture (including lactose), microcrystalline cellulose, trehalose, and calcium stearate. The mothers were instructed to take three tablets once a day after breakfast. All participants recorded a diary about the ingestion of the test tablets (only for the probiotic group), fermented foods, and medicines everyday throughout the study.

**Table 1:** Composition of the probiotic tablets.

**Nutrients**	**Probiotic tablet**
Energy (kcal)	1.2
Protein (g)	0–0.1
Fat (g)	0–0.1
Carbohydrates (g)	0.7
Sodium (mg)	0–2
*Lactobacillus casei* LC5 (CFU)	5 × 10^9^
*Bifidobacterium longum* BG7 (CFU)	5 × 10^9^
*Bacillus coagulans* SANK70258 (CFU)	2 × 10^8^

*Values are shown for the daily dose of the probiotic tablets (750 mg of three tablets per day)*.

### Collection of Breast Milk Samples

Mothers collected breast milk samples manually at 1–3 months postpartum after breastfeeding, using a breast pump (Yanase Waichi, Japan). Individual samples were collected in plastic tubes and stored at −80°C for further analyses.

### Measurements of TGF-β and IgA in Human Milk

Human milk levels of TGF-β1, TGF-β2, and IgA levels were measured via commercial enzyme-linked immunosorbent assay (ELISA) kits. TGF-β1 and TGF-β2 levels were measured with a Human TGF-beta 1 DuoSet and a Human TGF-beta 2 DuoSet, respectively (R&D Systems, Minneapolis, MN, USA). IgA levels were measured with a Human IgA ELISA Quantitation Set (Bethyl Laboratories Inc., Montgomery, AL, USA). All ELISAs were conducted according to the respective manufacturers' instructions. The detection limits were 31.2 pg/mL for TGF-β1 and TGF-β2, and 7.8 ng/mL for IgA.

### Data Analyses

Baseline characteristics were analyzed using the Mann–Whitney *U*-test. Clinical history was analyzed using the Pearson's χ^2^ test. Comparison of human milk TGF-β1, TGF-β2, and IgA levels between the various time points were performed using the Wilcoxon's signed rank test, and the *p*-values were adjusted by Bonferroni correction (α = 0.017). Correlations were assessed via the Spearman's correlation coefficient between TGF-β1, TGF-β2, and IgA levels in the breast milk of all subjects (*n* = 60) at 1 and 3 months postpartum. All statistical analyses were performed with SPSS Statistics 24 (IBM Inc., Chicago, IL, USA). Unless otherwise noted, *p* < 0.05 was considered statistically significant.

### Ethics

This study was carried out in accordance with the recommendations of Ethical Guidelines for Clinical Research (Ministry of Health, Labor and Welfare, Japan), and approved by the Ethics Committee of the Faculty of Medicine, University of Yamanashi (Yamanashi, Japan; receipt No. 1042, 2013). The protocol was approved by the Ethics Committee of the Faculty of Medicine, University of Yamanashi. All subjects gave written informed consent in accordance with the Declaration of Helsinki. This study was registered the University Hospital Medical Information Network Clinical Trials Registry (UMIN ID 000036059, 2019).

## Results

### Baseline Characteristics and Clinical Histories of the Study Participants

**Figure 1:**
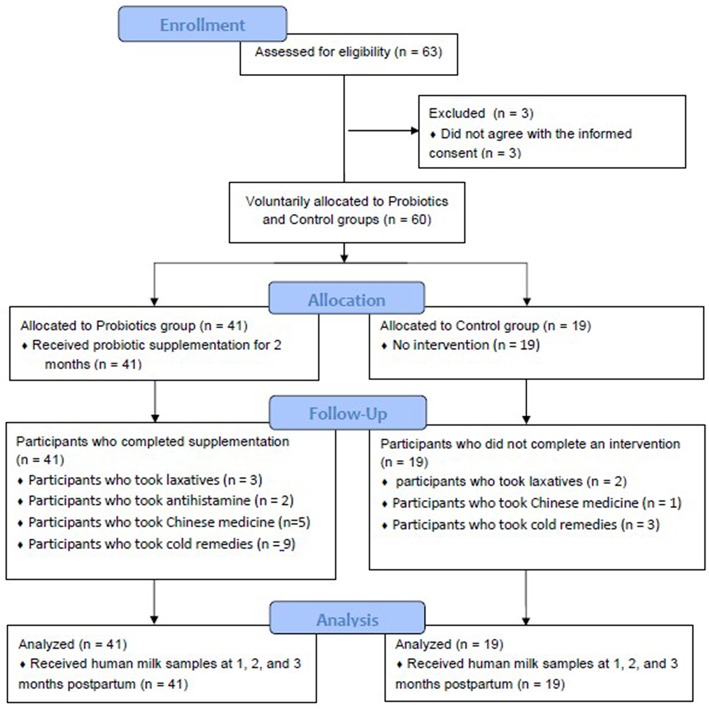
Participant disposition of this study.

The mothers in the probiotic and no supplementation control groups had similar baseline values for age, body mass index, gestational age, infant birth weight, days postpartum, breast milk TGF-β levels, and maternal allergic disease status at 1 month postpartum (Table 2). These indices did not differ significantly between the two groups, except for milk IgA, which was significantly lower in the supplementation group than that in the control group. While 41 mothers volunteered to take probiotics (the probiotic intervention group), there were only 19 mothers in the no supplementation control group. The participant disposition is shown in Figure 1. No adverse effects were reported during the study duration, according to the self-recorded diary in the supplementation periods. Some participants took medications such as laxatives (*n* = 3 in Probiotic, *n* = 2 in Control), antihistamines (*n* = 2 in Probiotic, none in Control), traditional Chinese medicines (*n* = 5 in Probiotic, *n* = 1 in Control), and cold remedies (*n* = 9 in Probiotic, *n* = 3 in Control), respectively.

**Table 2:** Baseline characteristics of the study participants at 1 month postpartum.

	**Probiotic group** **(*n* = 41)**	**Control group** **(*n* = 19)**	***P*-value**
Baseline characteristics			
Age (y)	33 (27–39)	33 (23–43)	0.981
Height (cm)	159 (150–169)	161 (154–168)	0.105
Weight (kg)	54 (45–63)	53 (46–61)	0.633
Gestational age (w)	39 (37–41)	39 (37–41)	0.775
Infant birth weight (g)	3110 (2,642–3,578)	3083 (2,802–3,274)	0.415
Days postpartum (d)	44 (34–48)	42 (32–52)	0.583
Levels of cytokines in human milk			
TGF-β1 (pg/mL)	164.0 (131.2–196.8)	180.7 (131.7–229.6)	0.460
TGF-β2 (pg/mL)	653.2 (473.4–833.1)	688.5 (423.8–953.2)	0.422
IgA (μg/mL)	507.8 (450.6–564.9)	605.0 (511.0–699.0)	0.030
Clinical history of allergies			
Any	41/41 (100%)	19/19 (100%)	1.000
Asthma	3/41 (7%)	3/19 (16%)	0.370
Atopic dermatitis	16/41 (39%)	6/19 (32%)	0.578
Allergic rhinitis	34/41 (83%)	13/19 (68%)	0.578

*Values are shown as median (interquartile range) except for clinical history of allergies, which is shown as proportion (%). BMI, body mass index; TGF, transforming growth factor; IgA, immunoglobulin A*.

### TGF- β1, TGF- β2, and IgA Levels in Breast Milk

**Figure 2:**
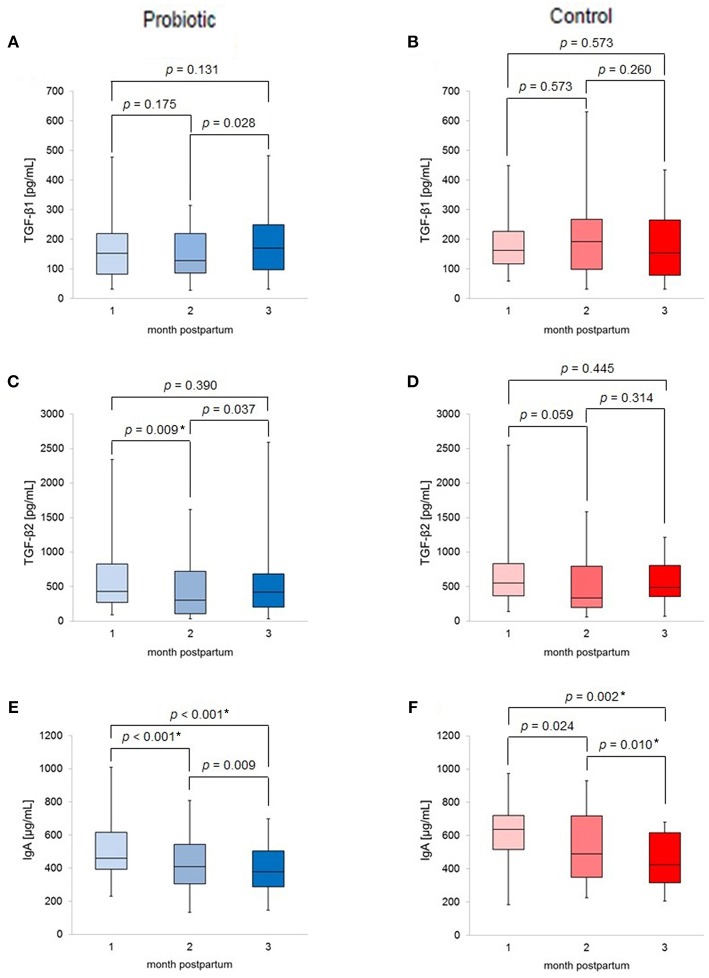
Box-plots of **(A)** TGF-β1 levels of probiotic group; **(B)** TGF-β1 levels of control group; **(C)** TGF-β2 levels of probiotic group; **(D)** TGF-β2 levels of control group; **(E)** IgA levels of probiotic group; **(F)** IgA levels of control group; labeled p-values indicates *p < 0.017 (significant).

Figure 2 shows the changes in TGF-β1, TGF-β2, and IgA levels in the breast milk. Median IgA levels decreased with increasing time in both groups. In contrast, median TGF-βs levels showed different patterns in the time course between the two groups. In the probiotic group, median TGF-β1 levels increased from 2 to 3 months postpartum (*p* = 0.028). For TGF-β2, median levels significantly decreased from 1 to 2 months postpartum (*p* < 0.017), followed by a tendency to increase from 2 to 3 months (*p* = 0.037). However, these changes were not observed in the control group.

**Figure 3:**
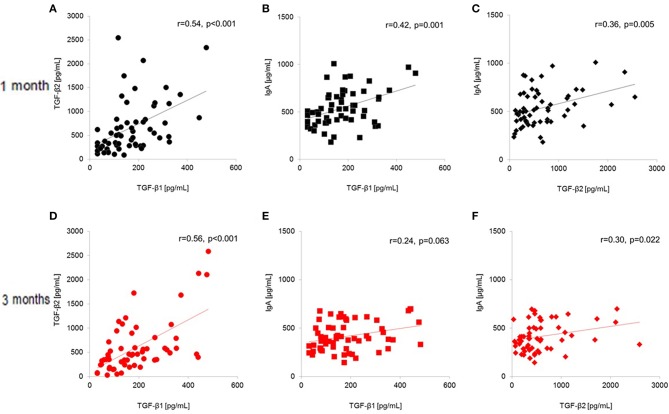
Total-population (*n* = 60) correlations between human milk TGF-β1, TGF-β2, and IgA **(A–C)** at baseline (1 month postpartum) and **(D–F)** at the end of the intervention (3 months postpartum). **(A)** TGF-β1 and TGF-β2 at 1 month postpartum; **(B)** TGF-β1 and IgA at 1 month postpartum; **(C)** TGF-β2 and IgA at 1 month postpartum; **(D)** TGF-β1 and TGF-β2 at 3 months postpartum; **(E)** TGF-β1 and IgA at 3 months postpartum; **(F)** TGF-β2 and IgA at 3 months postpartum.

Figure 3 shows correlations between TGF-β and IgA levels in the breast milk of all subjects (*n* = 60) at baseline and at the end of the intervention. Levels of TGF-β1, TGF-β2, and IgA were significantly and positively correlated at baseline (1 month postpartum; TGF-β1 and TGF-β2: *r* = 0.54, *p* < 0.001, TGF-β1 and IgA: *r* = 0.42, *p* = 0.001, TGF-β2 and IgA: *r* = 0.36, *p* = 0.005). At the end of the study (3 months postpartum; TGF-β1 and β2: *r* = 0.56, *p* < 0.001, TGF-β1 and IgA: *r* = 0.24, *p* = 0.063, TGF-β2 and IgA: *r* = 0.30, *p* = 0.022), the correlation between TGF-β1 and IgA was no longer significant while the other correlations remained significant.

In addition, the inter-group differences and the time-dependent decrease in IgA levels were also significant as shown using a mixed linear model for the supplementation time and interaction effects (data not shown).

## Discussion

This study was an open-label, parallel-group pilot trial to evaluate the effect of probiotic supplementation in lactating women on breast milk TGF-β and IgA levels during the first 3 postpartum months. Previous studies have investigated sufficiently the long-term efficacy of probiotics in pregnant and lactating mothers as well as infants, including 4 months of supplementation with a single *Lactobacillus* strain (2 × 10^10^ CFU/d) (6) and 7 months of supplementation with two *Lactobacillus* and *Bifidobacterium* strains (6 × 10^9^ and 9 × 10^9^ CFU/d) (7). The short-term efficacy study assessed TGF-β and IgA levels after supplementation to mothers at 3 months postpartum for 30 days with a symbiotic agent containing seven strains of probiotic species (total 2 × 10^8^ CFU/d) and fructo-oligosaccharides (394 mg/d) (8). Based on the self-reported diary current this study, there was no diarrheal stool in the probiotic group, even though laxatives were prescribed in three participants. This suggests that this particular probiotic combination is not likely to inducing gastrointestinal problems such as diarrhea.

However, as differences in intervention specifics and study settings may influence outcomes, the immunological effects of different probiotic combinations should be investigated individually. Species and strains often have disparate effects on biology. A mixture containing *L. casei* LC5 reduced NO production from the murine RAW264.7 cell line with bacterial lipopolysaccharide, indicating that LC5 involves anti-inflammatory activity (18). Furthermore, *B. longum* BG7 suppresses H. pylori-induced IL-8 production in human gastric cell lines (19), implying that BG7 can affect cytokine production induced by bacterial infection. Moreover, *B. coagulans* SANK70258 improves the intestinal environment in healthy adults, as evidenced by an increase in bifidobacteria and a decrease in *Clostridium perfringens* (17). Given this background, these bacterial strains have displayed a promising anti-inflammatory response to bacterial infections, as well as an improvement of the intestinal environment. Thus, we studied a combination of these three species. Although the safety and tolerability of probiotics have been evaluated individually, no apparent adverse effect was observed during the study in terms of a mixture of probiotics. Our findings would support the tolerability of probiotics in a mixture.

When compared to previous similar studies, we chose a medium-length study duration of 2 months, commencing at 1 month postpartum. Lactating mothers with a history of allergic disease voluntarily elected to take a probiotic-only intervention consisting of three strains, *L. casei* LC5, *B. longum* BG7, and *B. coagulans* SANK70258 (5 × 10^9^, 5 × 10^9^, and 2 × 10^8^ CFU/d, respectively). Approximately two-thirds of mothers voluntarily decided to participate in the probiotic intervention group, indicating that most lactating mothers with a history of allergy perceived the probiotics as acceptable. High adherence (91.5%) to the supplementation protocol is reflected by the lack of participant drop-out. However, the study design might have induced unintended effects, resulting in a reduction in the study.

Table 2 shows baseline characteristics and clinical histories of the study participants. These factors may affect the cytokine levels of human milk. In previous study, elevated maternal BMI was significantly associated with increased levels of some kind of cytokines in their milk (20). In this study, these factors showed similar values between the two groups except for IgA.

Figure 2 summarizes the changes in breast milk TGF-β1, TGF-β2, and IgA levels throughout the study. A recent study reported that the lactation change of TGF-β1 levels in human milk might be associated with allergic disease (21). The results of the present study are similar to or lower than those of Morita et al. (21) and comparable to those of Tomicic et al. (22). Although any differences could be attributed to differences in the cohorts, the TGF-βs levels in this study are within an appropriate range at 1–3 months postpartum. Moreover, median IgA levels decreased with the time of lactation in both groups; this seems to be the natural time course, as the protein level in human milk decreases with increased time of lactation (23).

In addition, using a mixed linear model for the supplementation, time and interaction effects, the inter-group differences and the time-dependent decrease in IgA levels were also significant (data not shown). Interestingly, TGF-β did not show this trend; the changes were different between the probiotic and control group, although there was no significant difference in median TGF-β levels in the two groups at the same month postpartum. As shown in Figure 2, the apparently the different time course trend between the two groups may be a result of the unequal sample sizes. Previous studies have reported an increase in breast milk TGF-β2 levels without significant changes in TGF-β1 levels (6, 8). Moreover, the results of those studies differed from ours in that IgA levels in breast milk also increased (7). Additionally, findings concerning the effects of probiotic supplementation on breast milk composition have been inconsistent, with discrepancies in the reported results potentially attributable to the probiotic strains used, the sociological background of the participants, and other potential factors.

Since IgA levels were significantly different at the baseline in this study (Table 2), the different IgA levels might be attributed to the voluntarily selection of the probiotic supplementation and the factors associated to such selections. Another possibility is potential experimental errors in the immunological assays; however, we used commercially validated assay kits, and all tests were performed by a single laboratory worker in the same laboratory; therefore, the chance of error was low. In the present study, breast milk levels of TGF-β1, TGF-β2, and IgA were significantly positively correlated at 1 month postpartum (Figure 3), although only the correlation between TGF-β2 and IgA remained significant at the end of the intervention (3 months postpartum). These results imply that the correlation between TGF-β1 and IgA became non-significant by the end of the intervention. A recent study reported that TGF-β1, TGF-β2, TGF-β3, and IgA are significantly correlated in colostrum (24). In particular, TGF-β3 showed the highest correlation among TGF-β isoforms, but we did not measure TGF-β3 in human milk. Consequently, lactation-related changes in these components should be considered in discussing the effect of nutritional intervention on human milk component levels, regardless of the study duration.

The limitations of the present study include potential placebo effects on TGF-β1, TGF-β2, and IgA levels in breast milk, as participants self-selected their intervention status. Given the environmental and socioeconomic factors affecting the selection between the two arms, immunological backgrounds associated with these factors might affect the immune components levels (25–27). Previous studies have reported that TGF-β and IgA levels in human milk differ with respect to the maternal environment factors such as ethnic-related factors, geographical location, dietary pattern, socioeconomic situation and psychosocial condition. Moreover, the effects of different sample sizes could also affect the outcomes. In terms of dietary factors, we assessed the possible association between TGF-β and IgA levels in human milk and the intake of fermented foods during the study. However, no significant associations were observed between the frequency in the intake of fermented foods and these compounds in human milk (data not shown). These effects may affect the validity of the correlations observed; therefore, it should be considered in interpreting the study results. To determine the comprehensive effects on human milk from pregnancy to the end of weaning, a randomized, placebo-controlled trial is needed. In addition, a longer follow-up of infants could be used to investigate longer term safety outcomes.

In conclusion, in this open-labeled pilot trial, we demonstrated that probiotic supplementation was well-tolerated in lactating mothers and the levels of TGF-β and IgA were affected by complex factors in their breast milk. Thus, probiotic supplementation in lactating women requires further investigations in terms of the dietary management of allergic outcomes in infants.

## Author Contributions

Conceptualization, methodology, and resources HF, SW-M, and TN. Validation, TT and HU. Measurements of TGF-β and IgA, KI. Data Analyses, TT, HF, KI, and AN. Investigation, HF, SW-M, KI, and AN. Writing and original draft preparation, HF. Writing-review and editing, TT, HF, HU, and TK. Visualization, TT, HF, and HU. Supervision, HU and TN. Project administration, TK and TN. Funding acquisition, TK and TN. All authors contributed to manuscript revision, read, and approved the submitted version.

### Conflict of Interest Statement

TT, HF, HU, SW-M, TK, and TN are employees of Bean Stalk Snow Co., Ltd. KI and AN received an honorarium from Bean Stalk Snow Co., Ltd.

## Abbreviations

ELISA: enzyme-linked immunosorbent assay
IgA: immunoglobulin A
TGF-β: transforming growth factor-β.
